# Egg parasitoids of *Arboridia apicalis* (Nawa, 1913) (Hemiptera, Cicadellidae), a leafhopper pest of grapevines in Japan, with description of a new species of *Anagrus* Haliday, 1833 (Hymenoptera, Mymaridae)

**DOI:** 10.3897/zookeys.945.51865

**Published:** 2020-07-03

**Authors:** Serguei V. Triapitsyn, Tetsuya Adachi-Hagimori, Paul F. Rugman-Jones, Natsuko Kado, Nobuo Sawamura, Yutaka Narai

**Affiliations:** 1 Department of Entomology, University of California, Riverside, California, USA University of California Riverside United States of America; 2 Organization for Promotion of Tenure Track, University of Miyazaki, Miyazaki, Japan University of Miyazaki Miyazaki Japan; 3 Shimane Agricultural Technology Center, Izumo, Shimane, Japan Shimane Agricultural Technology Center Izumo Japan

**Keywords:** *Aphelinoidea* sp., egg parasitoid, grapevine pest, identification key, natural enemy, *Oligosita* spp., taxonomy

## Abstract

Several species of egg parasitoids (Hymenoptera: Mymaridae and Trichogrammatidae) of the leafhopper pest of grapevines in Japan, Arboridia (Arboridia) apicalis (Nawa) (Hemiptera, Cicadellidae), were reared and identified for the first time. Using a combination of genetic and morphological evidence, Anagrus (Anagrus) arboridiae Triapitsyn & Adachi-Hagimori, **sp. nov.** (Mymaridae) is described and illustrated from Honshu Island (Shimane Prefecture) and Kyushu Island (Miyazaki Prefecture). It is shown to be different from Anagrus (Anagrus) japonicus Sahad and *A. flaviapex* Chiappini & Lin, to which it is most similar; the latter species was originally described from China and is newly recorded here from Okinawa Island, Japan. Mitochondrial and nuclear ribosomal DNA sequence data provide clear evidence for the separation of *A. arboridiae* from *A. flaviapex*, *A. japonicus*, and some other members of the Anagrus (Anagrus) atomus (L.) species group. Two other species of *Anagrus* Haliday, A. (Anagrus) avalae Soyka and *A. atomus*, are also identified in Japan from eggs of the leafhoppers *Edwardsiana ishidae* (Matsumura) and Eurhadina ? betularia Anufriev, respectively. An updated key to females of the Japanese species of *Anagrus* is given. *Oligosita pallida* Kryger (a new record for Japan), *Oligosita* sp., and an Aphelinoidea (Aphelinoidea) sp. (Trichogrammatidae) were the other, although much less abundant, apparent egg parasitoids of *A. apicalis* in Shimane Prefecture, mainly in non-organic vineyards.

## Introduction

**Figure 1.**
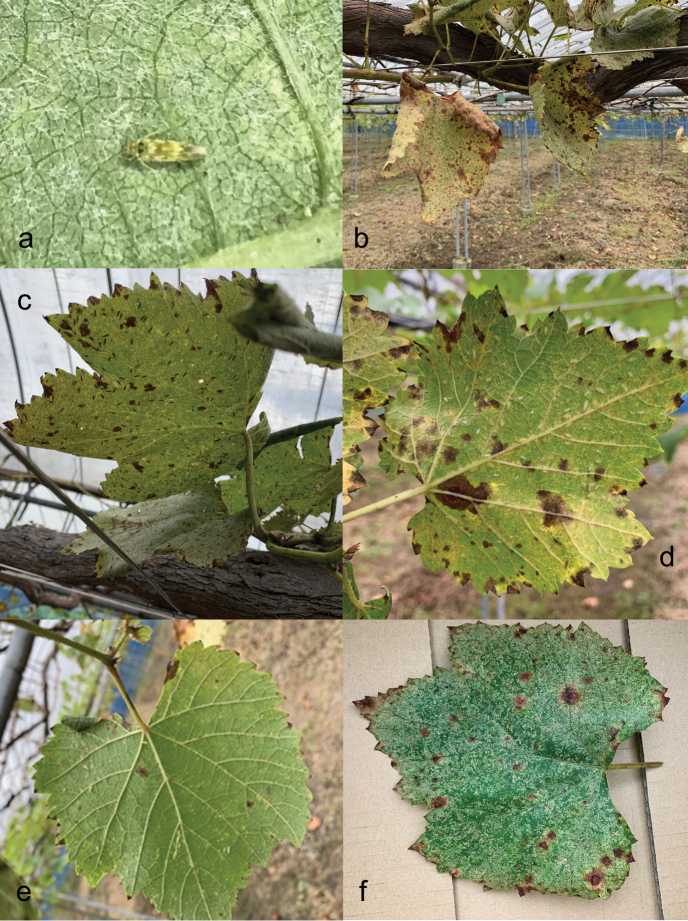
Arboridia (Arboridia) apicalis and its damage to cultivated grapevines in Japan **a** adult (Takayama, Gifu Prefecture, Honshu Island) **b** heavy damage to grape leaves in a covered vineyard (Shimane Prefecture, Honshu Island, also **c–f**) **c** numerous adults on the underside of a grape leaf **d** nymphs on the underside of a grape leaf **e** light damage to a grape leaf by a few nymphs **f** heavy damage to a grape leaf.

The leafhoppers Arboridia (Arboridia) apicalis (Nawa) (Fig. 1a) and A. (Arboridia) suzukii (Matsumura) (Hemiptera, Cicadellidae) have been recorded in Japan as pests of cultivated grapes, *Vitis* spp. (Vitaceae) (Tanaka et al. 1986; Sakagami 2003; Yamada 2003). Feeding by the leafhopper adults and nymphs causes stippling, a characteristic damage to the grape leaves (Fig. 1b–f); also, when abundant, particularly in the organic vineyards on Honshu Island, adult *A. apicalis* are a significant nuisance to the pickers at harvest by getting into peoples’ faces. Both *A. apicalis* and *A. suzukii* are native to the Eastern Palaearctic and Northeastern Oriental regions, with more or less similar distributions: that of *A. apicalis* includes Japan (throughout), Korean Peninsula, Far East of Russia, mainland China and Taiwan (Guglielmino et al. 2012; Song and Li 2013, 2015; Oh et al. 2015; Dmitriev 2019) while *A. suzukii* occurs in Japan (Honshu, Kyushu, and Shikoku Islands), Korean Peninsula, Far East of Russia, mainland China, and Taiwan (Oh et al. 2015; Dmitriev 2019). *Arboridia apicalis* is a polyphagous leafhopper feeding on various deciduous trees and vines, such as cherry, grape, hawthorn, apple, pear, peach, mulberry, maple (Yamada 2003; Guglielmino et al. 2012; Song and Li 2015; Dmitriev 2019). The known host plants of *A. suzukii*, besides grape, are apple, pear, Manchurian cherry (Dmitriev 2019), and also Japanese chestnut; this leafhopper is a pest in the Japanese vineyards mainly in Kyushu Island, causing damage particularly to leaf edges whereas *A. apicalis* damages the entire leaf more uniformly (Yamada 2003, illustrated by Sakagami (2003)). Both species overwinter as adults in dropped leaves, weeds, cracks in bark, and under cover of buildings. On Honshu Island, *A. apicalis* has three generations per year, although, in greenhouses, it develops earlier and can have four generations per year (Yamada 2003).

Egg parasitoids of *Arboridia* spp. have been unknown in Japan, yet elsewhere several species of Mymaridae and Trichogrammatidae (Hymenoptera: Chalcidoidea) were reported from eggs of other species of this genus (Noyes 2019). In particular, Anagrus (Anagrus) turpanicus Triapitsyn & Hu was described recently from eggs of the invasive leafhopper pest of grapes in Xinjiang Uyghur Autonomous Region of China, *Arboridia kakogawana* (Matsumura) (Hu and Triapitsyn 2016). Interestingly, *A. kakogawana* is native to Japan but it has not been reported there as a pest of cultivated grapes. One of the possible explanations for that, requiring further investigation, could be a possibility that the holotype female of *A. kakogawana*, collected (without indication of a host plant association) in Kakogawa (near Akashi), Hyogo Prefecture, Honshu Island (Matsumura 1932), might not be conspecific with the males from Korea, on whose genitalic characters the current recognition of this species is based upon (Dworakowska 1970). According to our observations, eggs of *A. apicalis* are laid singly, embedded in the soft tissues of grapevines, such as leaf veins. In the commercial vineyards of Japan, many of which are covered from at least above to control temperature and humidity, grape leafhoppers are usually controlled by insecticides (e.g., Arai and Toyama 2018) so finding organic vineyards, which are not common, was essential to the success of this survey. Because, according to the personal observations of the first author in other countries, grape leafhoppers from several genera, including *A. kakogawana*, prefer to feed on the leaves inside the vines, where it is more shady, it appears that the practice of covering the vines in many Japanese vineyards (and thus providing favorable shady conditions) might enhance damage to the entire vine, including peripheral foliage.

As the first step towards the establishment of a biological control-based integrated pest management (IPM) of these grape leafhoppers in Japan, we identified egg parasitoids of *A. apicalis* collected mainly from organic vineyards.

## Materials and methods

### Specimen collection

Both adults and nymphs of *A. apicalis* were collected in Japan by sweeping from the grapevines at several vineyards in Shimane Prefecture, Honshu Island, as well as from the organic vineyard in Aya, Miyazaki Prefecture, Kyushu Island (26°06'38"N, 129°41'16"E, 52 m). *Arboridia suzukii* was not present in these sites. Parasitized eggs, which turn dark orange as the parasitoid larva develops and then pupates, were documented by dissections in Petri dishes of the heavily leafhopper-infested grape leaves from the organic Oku-Izumo vineyard (Black Olympia cultivar) in Unnan, Shimane Prefecture. In early October 2019, leaves and vines infested with *A. apicalis* were collected from one organic and two non-organic vineyards in Shimane Prefecture and also in the above-mentioned organic vineyard in Miyazaki Prefecture; these were placed in tightly taped carton boxes. To collect adult parasitoids and host leafhoppers (both adults and nymphs), two clear glass vials were inserted through the holes into each of the boxes, one on the top and the other on the side near the top of the box, the latter to maximize exposure to daylight coming through the window. The vials were also exposed to a constant light source in the laboratory, and the emerging individuals were collected in 80% ethanol at least once almost daily (except for some weekends) for at least 30 days. Both host leafhoppers and parasitoids were sorted to morphospecies and identified by the first author at the second author’s laboratory at the University of Miyazaki Kibana Campus, Miyazaki City. The parasitoids were stored at -20 °C until they were shipped to the first author’s laboratory at the Entomology Research Museum, University of California at Riverside, California, USA (UCRC). These specimens were used both for molecular analyses and taxonomic studies (as type material of the new species of *Anagrus* Haliday described below).

Voucher specimens of the grape leafhoppers resulting from this study are deposited in the insect collections of the Entomological Laboratory, Faculty of Agriculture, Kyushu University, Fukuoka, Japan (**ELKU**), Illinois Natural History Survey, University of Illinois at Urbana-Champaign, Champaign, Illinois, USA (**INHS**) and the **UCRC**. Their identities were confirmed by Dmitry A. Dmitriev (INHS).

### Taxonomic studies

Morphological identifications of the *Anagrus* sp., made by the first author, were based mainly on females because males of many species of *Anagrus* are often similar and difficult to determine beyond a species group.

For the taxonomic description of the new species, the morphological terms of Gibson (1997), with some modifications made by Triapitsyn (2015), were used. All measurements (as length or length and width for the wings) are given in micrometers (µm). Abbreviations used in the description and key are:

**F** funicle segment of the female antenna or flagellomere of the male antenna;

**mps** multi-porous plate sensillum or sensilla on the antennal flagellar segments (= longitudinal sensillum or sensilla, or sensory ridge(s)).

Specimens from ethanol were dried using a critical point drier, then point-mounted and labeled. Selected specimens were dissected and slide-mounted in Canada balsam. Slide mounts were examined under a Zeiss Axioskop 2 plus compound microscope (Carl Zeiss Microscopy, LLC, Thornwood, New York, USA) and photographed using the Auto-Montage system (Syncroscopy, Princeton, New Jersey, USA). Photographs were retouched where necessary using Adobe Photoshop (Adobe Systems, Inc., San Jose, California, USA).

Specimens of the parasitoids examined are deposited in the collections with the following acronyms:

**CNC** Canadian National Collection of Insects, Arachnids and Nematodes, Ottawa, Ontario, Canada;

**ELKU** Entomological Laboratory, Faculty of Agriculture, Kyushu University, Fukuoka, Japan;

**UCRC** Entomology Research Museum, Department of Entomology, University of California, Riverside, California, USA.

### DNA extraction, amplification, and sequencing

DNA was extracted from two individual female wasps of the new species described herein using the “HotSHOT” method of Truett et al. (2000), in a total volume of 80 µL. This non-destructive method allowed for the recovery and slide-mounting of each specimen following extraction; each slide was then labeled with the assigned P. F. Rugman-Jones’ primary molecular voucher PR number and UCRC database UCRC ENT number. The polymerase chain reaction (PCR) was employed to amplify the “barcoding” region of the mitochondrial cytochrome c oxidase subunit I gene (COI) and the internal transcribed spacer 2 (ITS2) region of nuclear ribosomal RNA (rRNA). Amplification and sequencing of the COI were performed using the same protocols as described in Triapitsyn et al. (2018, 2019a, b) for some other species of *Anagrus*. It was only possible to sequence the ITS2 after cloning the amplicon as described in Triapitsyn et al. (2019b). For each specimen, two insert-positive clones were subsequently sequenced.

In addition to the specimens of the newly described species, the same methods were used to extract, amplify, and sequence the DNA from two individual females of Anagrus (Anagrus) japonicus Sahad, which represent the voucher specimens of the study by Adachi-Hagimori et al. (unpublished) (P. F. Rugman-Jones’ molecular vouchers PR19-501 [UCRC_ENT 005517343] and PR19-502 [UCRC_ENT 005517344]), and also from one female of A. (Anagrus) flaviapex Chiappini & Lin (data given below under “Comments”). Both species belong to the *atomus* species group of the nominate subgenus of *Anagrus*; all three specimens were recently collected in Okinawa Island, Ryukyu Islands, Japan.

All sequences generated in this study were deposited in GenBank (Benson et al. 2008).

### Genetic analysis

The COI sequences generated from the five specimens in this study (GenBank accessions MT396446–MT396450) were combined with 45 others retrieved from GenBank, each of which was a unique haplotype identified in three earlier studies of members of the *Anagrus* ‘*atomus*’ species complex, which is altogether comprised of six nominal species (Zanolli et al. 2016 [34 haplotypes, KM677212–KM677245]; Nugnes et al. 2017 [11 haplotypes from KX691520–KX691551]). One further sequence, from A. (Anagrus) incarnatus Haliday [MK024811], a member of the *incarnatus* species group, was also retrieved for use as an outgroup, and the combined sequence data were subsequently aligned using MAFFT version 7.050 (Katoh and Standley 2013) and the Q-INS-i algorithm with default settings. This resulted in an aligned COI dataset containing 51 terminal taxa and 587 nucleotide positions. The only gaps in the matrix were the result of 147 missing bases at the 5’ end of many of the sequences retrieved from GenBank. Genetic variation among our sequences was estimated by calculating uncorrected p-distances between all possible sequence pairs, using MEGA version 6 (Tamura et al. 2013). Gapped positions (i.e. at the 5’ end) were removed for each sequence pair in the analysis. A neighbor-joining (NJ) tree based on those p-distances was subsequently constructed, again using MEGA. Branch support was estimated using a bootstrap procedure with 1000 replicates.

Since phylogenetic inference from ITS2 is typically problematic due to large interspecific differences that make alignment of this region difficult and somewhat ambiguous, ITS2 sequences were examined “by eye” to corroborate the differentiation of our specimens based on COI. Furthermore, BLAST searches of the NCBI database were performed to assess their similarity to other members of the ‘*atomus*’ species complex.

## Results

### Taxonomy

Mymaridae

#### Anagrus (Anagrus) arboridiae

Taxon classificationAnimaliaHymenopteraTrichogrammatidae

Triapitsyn & Adachi-Hagimori sp. nov.

760923B8-1D8C-5BDB-9B4A-8E64E25828B4

http://zoobank.org/915C3341-6BD3-4E69-A6FD-8B47FD77792E

Figures 2 , 3 , 4

##### Type material.

***Holotype*** ♀ (Fig. 3a, b, d), deposited in ELKU, on slide (Fig. 3c) labeled: 1. “Japan: Honshu Island Shimane Prefecture, Unnan Oku-Izumo vineyard, 35°17'20"N, 132°55'46"E, 155 m (organic Black Olympia table grapes heavily infested with *Arboridia apicalis* (Nawa) in a covered vineyard), leaves collected 4.x.2019, N. Kado, N. Sawamura, T. Adachi-Hagimori, S. V. Triapitsyn Emerged 6.x.2019, S. V. Triapitsyn”; 2. [magenta] “Anagrus (Anagrus) arboridiae Triapitsyn & Adachi-Hagimori HOLOTYPE ♀”; 3. “Det. by S. V. Triapitsyn 2019”; 4. [barcode database label/unique identifier] “UCRC [bold] UCRC_ENT 005517345”.

**Figure 2.**
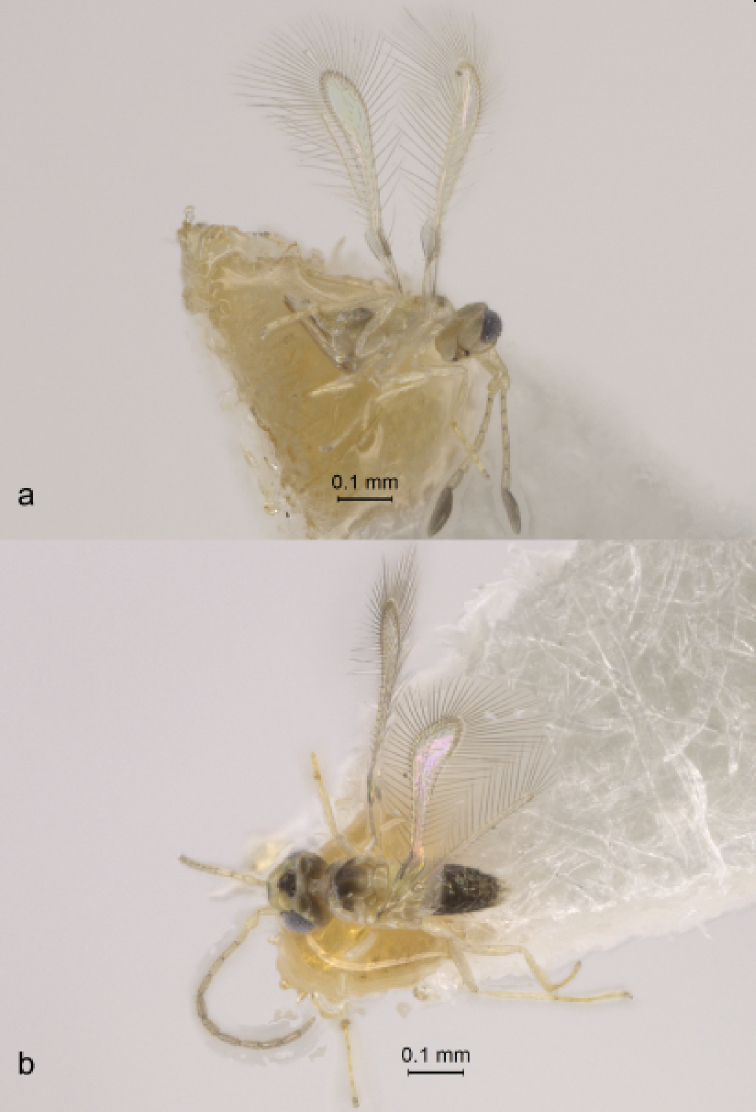
*Anagrus arboridiae* sp. nov. (paratypes from Unnan, Shimane Prefecture, Honshu Island, Japan) **a** habitus of female (lateral view) **b** habitus of male (dorsal view).

**Figure 3.**
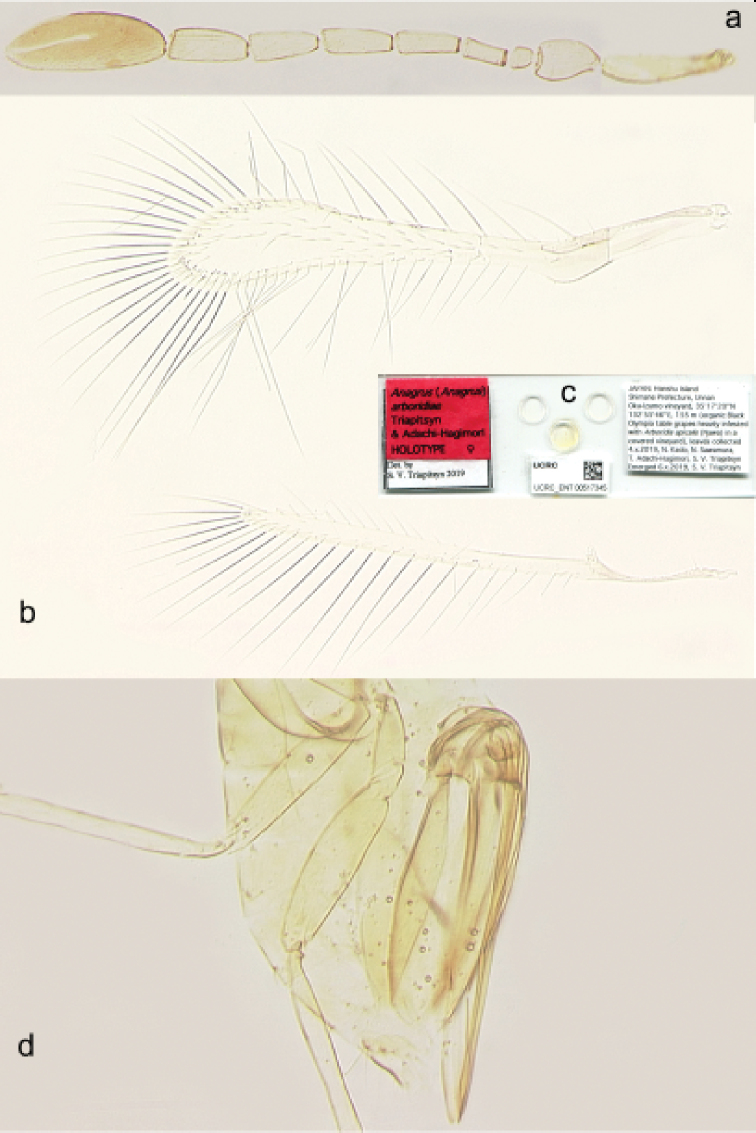
*Anagrus arboridiae* sp. nov. female (holotype) **a** antenna **b** fore and hind wings **c** slide **d** gaster.

***Paratypes*.** Japan: Honshu Island, Shimane Prefecture, Unnan, Oku-Izumo vineyard, 35°17'20"N, 132°55'46"E, 155 m (organic Black Olympia table grapes heavily infested with *A. apicalis* in a covered vineyard), collected 4.x.2019, N. Kado, N. Sawamura, T. Adachi-Hagimori, S. V. Triapitsyn, emerged from grape leaves: 5.x.2019, S. V. Triapitsyn [4 ♀♀, 6 ♂♂, ELKU, UCRC: 2 ♀♀, 5 ♂♂ on points and 2 ♀♀, 1 ♂ on slides (including 1 ♀ on slide, P. F. Rugman-Jones’ molecular voucher PR19-504, UCRC_ENT 005517353)]; 6.x.2019, S. V. Triapitsyn [15 ♀♀, 10 ♂♂, CNC, ELKU, UCRC: 8 ♀♀, 6 ♂♂ on points and 7 ♀♀, 4 ♂♂ on slides (including 1 ♀ on slide, P. F. Rugman-Jones’ molecular voucher PR19-503, UCRC_ENT 005517352)]. Kyushu Island, Miyazaki Prefecture, Higashimorokata, Aya, Kitamata, 32°00'32"N, 131°14'26"E, 86 m (Katsuki Wines LLC organic vineyard, Chardonnay wine grapes lightly infested with *A. apicalis* in a covered vineyard), leaves collected 30.x.2019, T. Adachi-Hagimori, S. V. Triapitsyn, emerged 6.xi.2019, S. V. Triapitsyn [1 ♀, 1 ♂ on slides, UCRC].

**Other (non-type) material examined.** Japan: Honshu Island, Shimane Prefecture, Unnan, Oku-Izumo vineyard, 35°17'20"N, 132°55'46"E, 155 m (organic Black Olympia table grapes heavily infested with *A. apicalis* in a covered vineyard), collected 4.x.2019, N. Kado, N. Sawamura, T. Adachi-Hagimori, S. V. Triapitsyn: sweeping on vines [1 ♀]; emerged from grape leaves: 5.x.2019, S. V. Triapitsyn [10 ♀♀, 10 ♂♂]; 6.x.2019, S. V. Triapitsyn [27 ♀♀, 22 ♂♂]; 7.x.2019, N. Kado [35 ♀♀, 29 ♂♂]; 8.x.2019, N. Kado [27 ♀♀, 18 ♂♂]; 9.x.2019, N. Kado [24 ♀♀, 12 ♂♂]; 10.x.2019, N. Kado [6 ♀♀, 6 ♂♂]; 11.x.2019, N. Kado [12 ♀♀, 7 ♂♂]; 13.x.2019, N. Kado [7 ♀♀]; 15.x.2019, N. Kado [1 ♀♀, 3 ♂♂]. Kyushu Island, Miyazaki Prefecture, Higashimorokata, Aya, Kitamata, 32°00'32"N, 131°14'26"E, 86 m (Katsuki Wines LLC organic vineyard, Chardonnay wine grapes lightly infested with *A. apicalis* in a covered vineyard), leaves collected 30.x.2019, T. Adachi-Hagimori, S. V. Triapitsyn: emerged 31.x.2019, S. V. Triapitsyn [1 ♂]; emerged 2.xi.2019, T. Adachi-Hagimori [1 ♂]; emerged 4.xi.2019, T. Adachi-Hagimori [2 ♀♀]; emerged 2.xi.2019, T. Adachi-Hagimori [1 ♂]; emerged 5.xi.2019, S. V. Triapitsyn [1 ♀]. All in 80% ethanol in a freezer [UCRC].

##### Diagnosis.

**Figure 4.**
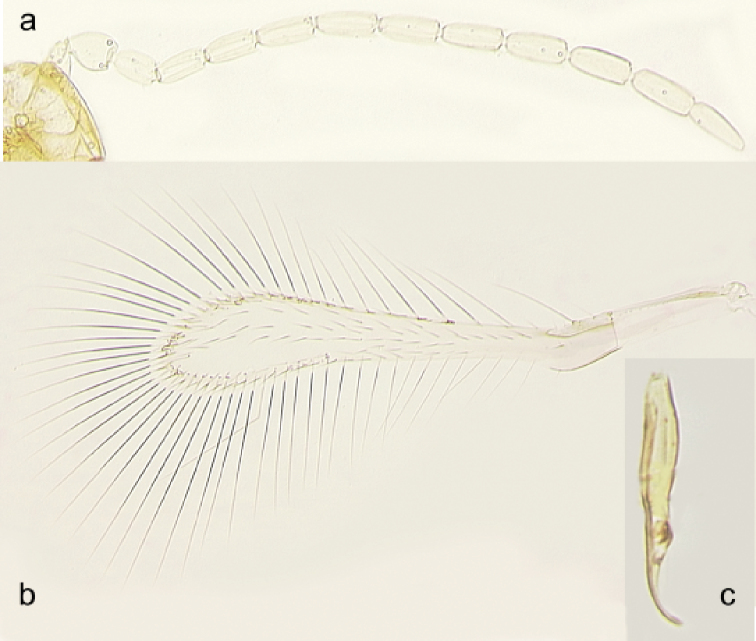
*Anagrus arboridiae* sp. nov. male (paratypes from Unnan, Shimane Prefecture, Honshu Island, Japan) **a** antenna **b** fore wing **c** genitalia (lateral view).

The new species is a member of the *atomus* species group of Anagrus (Anagrus) as defined by Chiappini et al. (1996). Female antenna (Fig. 3a) with mps on F3 (1), F4 (1), F5 (1 or 2), F6 (2), and clava (3); midlobe of mesoscutum without adnotaular setae; fore wing disc with a distinct subapical bare area (Figs 3b, 4b); ovipositor (Fig. 3d) 2.3–2.5× length of protibia. Male genitalia (Fig. 4c) with hooked digiti.

Morphologically, *A. arboridiae* is most similar to *A. flaviapex*, to which its female specimens with a more or less distinct bare area on the fore wing disc key in Triapitsyn (2015) and Li et al. (2018). Both taxa have one mps on F3 of the female antenna (Chiappini and Lin 1998). However, male genitalia of *A. flaviapex* have cone-shaped, straight digiti (Triapitsyn 1999) and body of the female is mostly brown (Triapitsyn 2015). Besides *A. arboridiae*, the three other known species within the nominate subgenus of *Anagrus* with hooked digiti of male genitalia and three mps on the clava of female antenna, the European *A. vilis* Donev and the Afrotropical *A. scassellatii* Paoli and *A. sensillatus* Viggiani & Jesu (Chiappini and Mazzoni 2000), were assigned to the *atomus* species group by them and also by Triapitsyn (2015). More recently, Nugnes et al. (2017) placed these, without providing much of a justification and based solely on the hooked shape of the digiti of male genitalia, in a separate *vilis* species group of Anagrus (Anagrus). However, based on other morphological features of both sexes *A. arboridiae* is not related to *A. vilis* and either of them are not related to the two aforementioned Afrotropical species. Moreover, in males of *A. japonicus* from Okinawa Island, shape of the digiti is of an intermediate state between the strongly hooked (such as in *A. arboridiae* and *A. vilis*) and the straight ones (like in most other species within the *atomus* species group): these are more or less cone-shaped in dorsoventral view but notably curved in lateral view (Adachi-Hagimori et al. unpublished). Because the hooked shape of the digiti is likely to have evolved independently in the different lineages within the *atomus* species group *sensu lato*, we agree with the conclusion made by Chiappini and Mazzoni (2000) that by itself this feature should not be used for defining separate species groups without support of any additional characters. Thus, we do not recognize the *vilis* species group proposed by Nugnes et al. (2017) as a separate entity from the *atomus* species group.

Females of *A. arboridiae* are also similar to those *A. japonicus* that sometimes have an mps on F3, but the former always have distinct light brown and brown patches on the mesoscutum and the basal gastral terga respectively, which the latter species lacks.

An updated key to females of the Japanese species of *Anagrus* is provided below, as the latest key by Triapitsyn et al. (2019a) is already outdated after the addition of four species herein which are new to the fauna of Japan.

##### Description.

**Female** (holotype and paratypes). Body length of dry-mounted, critical point-dried paratypes 400–500 µm, and of the slide-mounted paratypes 415–520 µm. Body (Fig. 2a) and appendages mostly pale yellow except funicle, occiput and almost all of mesoscutum (except posteriorly) light brown, transverse trabecula and stemmaticum dark brown, clava and two basal gastral terga brown; wings almost hyaline (slightly infumate). Antenna (Fig. 3a) with scape 3.4–4.1× as long as wide, with cross-ridges, 2.0–2.3× length of pedicel; F1 at most slightly longer than wide, almost half of pedicel length; F2 usually notably shorter than following funicular segments but occasionally approximately as long as F3 or even slightly longer (when F3 lacks mps), F4 usually slightly longer than F5 but occasionally subequal to F5, F6 the longest funicular segment; mps on F3 (usually one but often none), F4 (one or two); F5 (one), and F6 (two); clava with three rather short mps (just a little longer than half length of clava), 2.7–3.3× as long as wide, usually slightly longer (but occasionally approximately as long as) than combined length of F5 and F6. Midlobe of mesoscutum without adnotaular setae. Fore wing (Fig. 3b) 6.1–7.0× as long as wide, longest marginal seta 2.5–2.6× maximum wing width; distal macrochaeta ca. 4× length of proximal macrochaeta; disc with several rows of setae in addition to admarginal rows of setae (single complete row originating behind apex of venation and two or three irregular rows in the broadest part of disc), leaving a distinct subapical bare area at posterior margin. Hind wing (Fig. 3b) 21–25× as long as wide, longest marginal seta 7.4–9.0× maximum wing width; disc mostly bare except for an almost complete row of microtrichia along posterior margin and 1–3 additional microtrichia at apex. Ovipositor (Fig. 3d) extending anteriorly almost to mesophragma in slide-mounted specimens and at most barely exserted beyond apex of gaster posteriorly (by at most 0.06× total ovipositor length). Second valvifers (= external plates of ovipositor), e.g., Chiappini et al. (1996), each with 1 seta. Ovipositor 1.9–2.1× length of protibia (2.05× in the holotype).

***Measurements*** (µm) of the holotype (as length or length: width). Body: 545; mesosoma 197; gaster 251; ovipositor 212. Antenna: scape 75; pedicel 38; F1 13; F2 27; F3 39; F4 43; F5 42; F6 48; clava 100. Fore wing 463: 74; longest marginal seta 185. Hind wing 424: 20; longest marginal seta 148.

**Male** (paratypes). Body length of dry-mounted, critical point-dried paratypes 330–460 µm, and of the slide-mounted paratypes 400–520 µm. Body color mainly as in female except most of mesoscutum brown and most of gaster dark brown (light brown basally) (Fig. 2b); flagellum light brown. Antenna (Fig. 4a) with scape 2.6–3.2× as long as wide, F1 usually at least slightly shorter than following flagellomeres but occasionally either much shorter than F2 or approximately as long as F2. Fore wing (Fig. 4b) 5.9–6.4× as long as wide. Genitalia (Fig. 4c) length 86–90 µm; digiti hooked.

##### Etymology.

This new species is named after the host leafhopper genus.

##### Distribution.

Palaearctic region: Japan (Honshu and Kyushu Islands).

##### Host.

Cicadellidae: Arboridia (Arboridia) apicalis (Nawa). Only *A. apicalis* was present on the grapevines both in Aya, Miyazaki Prefecture and in Unnan, Shimane Prefecture, with any other leafhoppers being absent, so this host association of *A. arboridiae* is obvious.

##### Biology.

In the dissected parasitized eggs of *A. apicalis* in the organic vineyard in Unnan, Shimane Prefecture, *A. arboridiae* was observed to develop as a solitary endoparasitoid. Other aspects of its biology are unknown and thus would require further investigations.

##### Remarks.

The following species of *Anagrus* are newly recorded for the fauna of Japan.

Anagrus (Anagrus) flaviapex: Japan, Ryukyu Islands, Okinawa Prefecture, Okinawa Island, Itoman, Makabe, Okinawa Prefectural Agricultural Research Center (26°06'37.9"N, 129°41'16.1"E, 52 m), okra (organic experimental plot), 15–18.x.2019, S.V. Triapitsyn, T. Adachi-Hagimori, T. Uesato, Malaise trap [1 ♀, UCRC; P. F. Rugman-Jones’ molecular voucher PR19-505, UCRC_ENT 005517351]. This species has an Oriental and Eastern Palaearctic distribution but is known from different leafhopper and possibly also planthopper hosts (Chiappini and Lin 1998; Triapitsyn 1999, 2015).

The following dry-mounted specimens of *Anagrus* were found in T. Tachikawa’s collection during the first author’s visit of ELKU in October 2019 and consequently borrowed, some of them slide-mounted, and then identified. They represent new and interesting host associations for the respective taxa:

Anagrus (Anagrus) atomus (L.): Japan, Shikoku Island, Ehime Prefecture, Kihoku, 24.iii.1966, emerged 28.iii.1966 from eggs of Eurhadina ? betularia Anufriev (a tentative identification of the host per the original label in Japanese) [4 ♀♀, ELKU];

Anagrus (Anagrus) avalae Soyka: Japan, Honshu Island, Aomori Prefecture, Hirosaki City, vi.1964, R. Tsugawa, from eggs of *Edwardsiana ishidae* (Matsumura) [13 ♀♀, 4 ♂♂, ELKU].

### Key to females of the Japanese species of *Anagrus*

**Table:** 

1	Ocelli on a stemmaticum	**3**
–	Ocelli not on a stemmaticum (Anagrus (Anagrella) Bakkendorf)	**2**
2	F2 approximately 1.5× F1 length	**Anagrus (Anagrella) brevis Chiappini & Lin**
–	F2 at least 4.0× F1 length	**Anagrus (Anagrella) hirashimai Sahad**
3	Frenum with triangular paramedial plates widely separated from each other; metafemur short, less than 2× trochanter length, trochantellus incision almost half-way between coxa-trochanter and femur-tibia articulations (Anagrus (Paranagrus) Perkins)	**4**
–	Frenum with triangular paramedial plates very close to each other; metafemur long, more than 2× trochanter length, trochantellus incision ca. one-third way between coxa-trochanter and femur-tibia articulations (Anagrus (Anagrus) Haliday [*sensu sricto*])	**5**
4	Ovipositor projecting beyond apex of gaster by approximately 1/3 of its total length; ovipositor length: protibia length ratio at least 3.5	**Anagrus (Paranagrus) perforator (Perkins)**
–	Ovipositor not projecting or at most slightly projecting beyond apex of gaster; ovipositor length: protibia length ratio at most 2.5	**Anagrus (Paranagrus) optabilis (Perkins)**
5	Clava with 3 mps (*atomus* species group)	**6**
–	Clava with 5 mps (*incarnatus* species group)	**10**
6	Fore wing length: width ratio more than 10	**Anagrus (Anagrus) frequens Perkins**
–	Fore wing length: width ratio less than 10	**7**
7	Body light brown or brown (at most frenum, propodeum and apex of gaster yellow)	**8**
–	Body pale yellow, yellow, or greyish yellow (at most parts of mesoscutum and 2 basal gastral terga light brown or brown)	**9**
8	F3 without mps; gaster uniformly colored	**Anagrus (Anagrus) atomus (L.)**
–	F3 with 1 mps; 2 apical gastral terga contrastingly yellow	**Anagrus (Anagrus) flaviapex Chiappini & Lin**
9	Body pale yellow except mesoscutum mostly light brown and 2 basal gastral terga contrastingly brown	**Anagrus (Anagrus) arboridiae Triapitsyn & Adachi-Hagimori, sp. nov.**
–	Body yellow or greyish yellow, with 2 basal gastral terga concolorous with the rest of metasoma	**Anagrus (Anagrus) japonicus Sahad**
10	Midlobe of mesoscutum with adnotaular setae	**11**
–	Midlobe of mesoscutum without adnotaular setae	**12**
11	Body light yellow except mesoscutum partially a little darker; each external plate of ovipositor (second valvifer) with 2 setae	**Anagrus (Anagrus) avalae Soyka**
–	Body brown; each external plate of ovipositor with 3 setae	**Anagrus (Anagrus) subfuscus Foerster**
12	Fore wing approximately 6.3× as long as wide	**Anagrus (Anagrus) takeyanus Gordh**
–	Fore wing at least 7.0× as long as wide	**13**
13	F2 the longest funicular segment	**Anagrus (Anagrus) incarnatus Haliday**
_	F2 at least slightly shorter than following funicular segments	**Anagrus (Anagrus) rugmanjonesi Triapitsyn & Adachi-Hagimori**

### Trichogrammatidae

#### Oligosita pallida

Taxon classificationAnimaliaHymenopteraTrichogrammatidae

Kryger, 1919

00C0A465-C73B-5023-924B-C31FFF833682

Figure 5

Oligosita pallida Kryger, 1919: 318–319. 2 syntype ♀♀ [Zoological Museum, Natural History Museum of Denmark, University of Copenhagen, Copenhagen, Denmark (ZMUC)] (not examined). Type locality: Gentofte, Denmark.
Oligosita pallida Kryger: Viggiani 1987: 543–546 (synonymy, type material, diagnosis, distribution, host associations, illustrations); Liu and Li 2019: 1125 (key), 1126–1127 (taxonomic history, list of synonyms, distribution, hosts).
Oligosita sp.: Triapitsyn, 1998: 83 (reared from eggs of an Arboridia sp. in Turkmenistan).

##### Material examined.

Japan, Honshu Island, Shimane Prefecture: Izumo, Taisha (emerged from leaves of non-organic table grape infested with *A. apicalis* in a covered vineyard): 35°21'11"N, 132°40'59"E, 8 m, Shine Muscat grapes: collected 25.ix.2019, N. Kado, N. Sawamura, emerged 2–6.x.2019, S. V. Triapitsyn, N. Kado [2 ♂♂, UCRC]; collected 30.ix.2019, N. Kado, N. Sawamura, emerged 6.x.2019, S. V. Triapitsyn [1 ♀, UCRC]; collected 2.x.2019, N. Kado, Y. Narai, T. Adachi-Hagimori, S. V. Triapitsyn, emerged 3–5.x.2019, S. V. Triapitsyn, N. Kado [3 ♀♀, UCRC]. 35°21'21"N, 132°41'22"E, 3 m, Watanabe vineyard, Delaware grapes, collected 1.x.2019, N. Kado, N. Sawamura: emerged 3.x.2019, S. V. Triapitsyn, N. Kado [2 ♀♀, 1 ♂, UCRC]; emerged 4.x.2019, S. V. Triapitsyn, N. Kado [1 ♀, UCRC]. Unnan, Oku-Izumo vineyard, 35°17'20"N, 132°55'46"E, 155 m (organic Black Olympia table grapes heavily infested with *A. apicalis* in a covered vineyard), leaves collected 4.x.2019, N. Kado, N. Sawamura, T. Adachi-Hagimori, S. V. Triapitsyn, emerged 17.x.2019, N. Kado [1 ♀, UCRC].

##### Other material examined.

France, Gironde, Sainte Colombe, M. van Helden, in vineyards: 44°52'N, 00°02'W [3 ♀♀, UCRC]; Pitray, 44°54'N, 00°02'W [1 ♀, UCRC]. Iran: Isfahan, vi.2000, S. Hesami, from grape leafhopper [1 ♀, UCRC]. Khorasan, Fazd Village, 10.x.1994, J. Vafabaksh, from grape leaves [1 ♀, UCRC]. Turkmenistan, Ashgabat, 24.viii.1993, S. N. Myartseva, from eggs of Arboridia (Arboridia) hussaini (Ghauri) on grape [1 ♀, UCRC].

##### Distribution.

Palaearctic region: China (Liu and Li 2019), Croatia (Bakkendorf 1971 [for *Oligosita tominici* Bakkendorf, a synonym of *O. pallida* (Viggiani 1987)]), Czech Republic, Denmark, Hungary, Iran, Italy, Netherlands, Turkey, UK (Noyes 2019), France, Japan, and Turkmenistan (new records).

##### Hosts.

Cicadellidae: Arboridia (Arboridia) apicalis (Nawa), A. (Arboridia) hussaini (Ghauri) (Triapitsyn 1998 [as *Arboridia* sp. for *Oligosita* sp.]) (new records), as well as A. (Arboridia) adanae (Dlabola) (Viggiani 1987; Yiğit and Erkiliç 1987), A. (Arboridia) kermanshah Dlabola (Mostaan and Akbarzadeh 1995), *Edwardsiana rosae* (L.) (Viggiani 1987), Zygina (Zygina) eburnea Fieber (Bakkendorf 1971 [as *Erythroneura eburnea* for *O. tominici*]), and Zygina (Zygina) rhamni Ferrari (Viggiani 1987).

##### Remarks.

**Figure 5.**
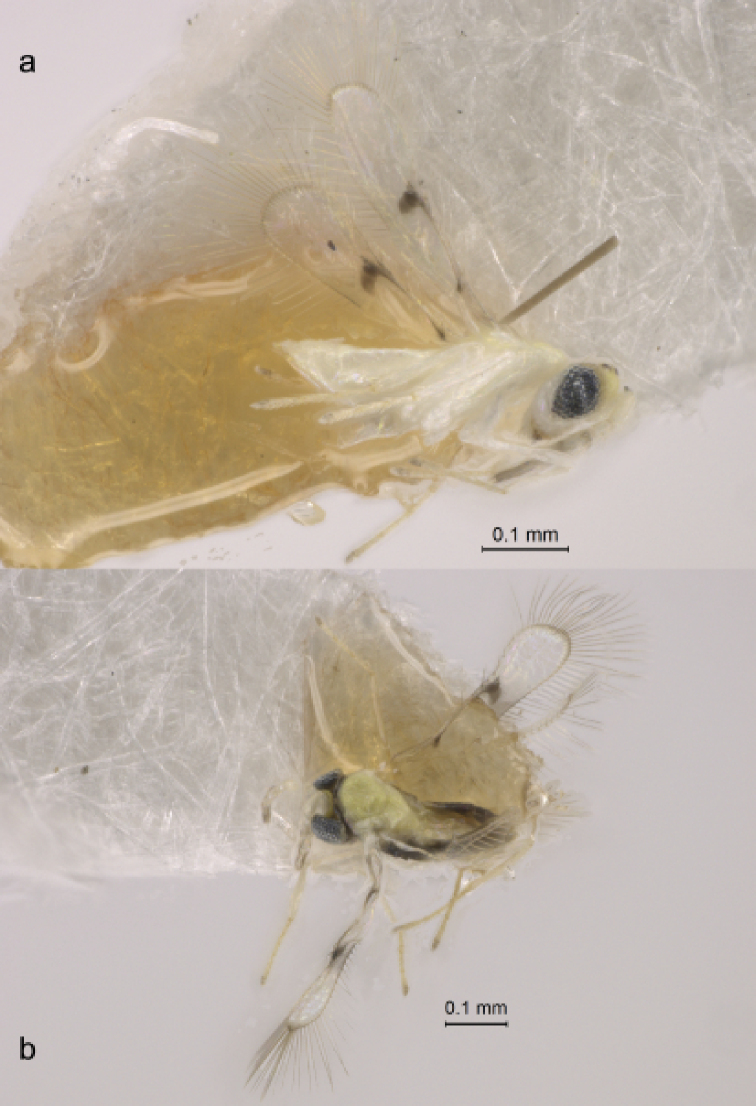
*Oligosita pallida* (Taisha, Izumo, Shimane Prefecture, Honshu Island, Japan) **a** habitus of female (lateral view) **b** habitus of male (dorsolateral view).

Females of this distinctive species can be recognized by the uniformly pale color of the body (Fig. 5a), while in males the sides of metasoma and sometimes mesosoma are dark (Fig. 5b). Other distinguishing features of both sexes include a rather long clava of the antenna and a characteristic fore wing with a distinctive dark spot behind the apex of venation (Fig. 5), as described and illustrated by Kryger (1919) and Viggiani (1987).

Other examined female specimens of an *Oligosita* sp. in UCRC, collected from grape leaves in Iran, have several small dark spots on the sides of metasoma; thus, we cannot positively identify them as *O. pallida* without availability of a thorough assessment of intraspecific variation of body color in this species.

#### Oligosita

Taxon classificationAnimaliaHymenopteraTrichogrammatidae

sp.

926507F7-6166-5B72-BF13-D7F95C35BCA7

Figure 6a

##### Material examined.

Japan, Honshu Island, Shimane Prefecture, Izumo, Taisha, 35°21'21"N, 132°41'22"E, 3 m, 5.x.2019, N. Kado, N. Sawamura, T. Adachi-Hagimori, S. V. Triapitsyn (sweeping upon leaves of non-organic Delaware table grapes infested with *A. apicalis* in a covered Watanabe vineyard) [1 ♀, UCRC].

##### Distribution.

Palaearctic region: Japan (Honshu Island).

##### Host.

Cicadellidae: Arboridia (Arboridia) apicalis (Nawa). This tentative host association will need to be confirmed by experimental work using sentinel eggs of this leafhopper.

##### Remarks.

**Figure 6.**
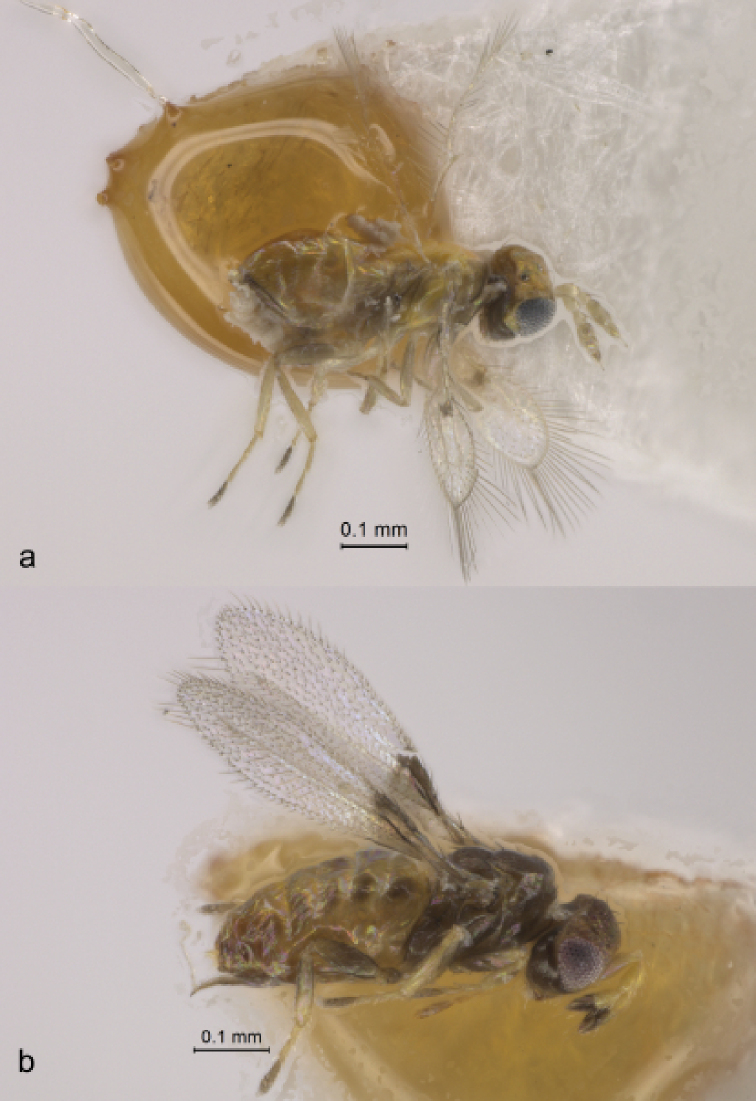
Habitus in lateral view **a***Oligosita* sp. female (Taisha, Izumo, Shimane Prefecture, Honshu Island, Japan) **b**Aphelinoidea (Aphelinoidea) sp. male (Unnan, Shimane Prefecture, Honshu Island, Japan).

Because taxonomy of the genus *Oligosita* Walker is in flux and no working keys are available, we could not positively identify this specimen to the species. Its body color is more or less light brown except dorsum of mesosoma is mostly yellow (Fig. 6a).

#### Aphelinoidea (Aphelinoidea)

Taxon classificationAnimaliaHymenopteraTrichogrammatidae

sp.

9CEEC82A-8881-5BDA-B831-3061F922F32A

Figure 6b

##### Material examined.

Japan, Honshu Island, Shimane Prefecture, Unnan, Oku-Izumo vineyard, 35°17'20"N, 132°55'46"E, 155 m, leaves of organic Black Olympia table grapes heavily infested with *A. apicalis* in a covered vineyard collected 4.x.2019, N. Kado, N. Sawamura, T. Adachi-Hagimori, S. V. Triapitsyn, emerged 5.x.2019, S. V. Triapitsyn [1 ♂, UCRC].

##### Distribution.

Nearctic region: USA (Nebraska); Palaearctic region: Japan (Honshu Island).

##### Host.

Cicadellidae: Arboridia (Arboridia) apicalis (Nawa). This tentative host association will need to be confirmed by experimental work using sentinel eggs of this leafhopper.

##### Remarks.

This specimen is similar to Aphelinoidea (Aphelinoidea) waterhousei (Blood & Kryger) which belongs to the *semifuscipennis* species group of the nominate subgenus of the genus *Aphelinoidea* Girault, formerly placed in the synonymized subgenus Aphelinoidea (*Diaclava* Blood & Kryger) (Triapitsyn 2018). Like *A. waterhousei* from England, UK, known from the single male specimen, the one from Japan (Fig. 6b) has apical segment of the clava contrastingly darker than the light-colored basal segment, and the marginal vein of its fore wing is notably thickened while the stigmal vein is short, inconspicuous. But because the fore wing of the specimen from Japan is notably wider and its marginal setae are relatively shorter (ca. one-third of the greatest fore wing width), it is unlikely to be *A. waterhousei*; rather, it is more similar to the unidentified male specimen from Nebraska, USA which has the same features, as illustrated by Triapitsyn (2018: 78, figs 93–97).

### Molecular analyses

**Figure 7.**
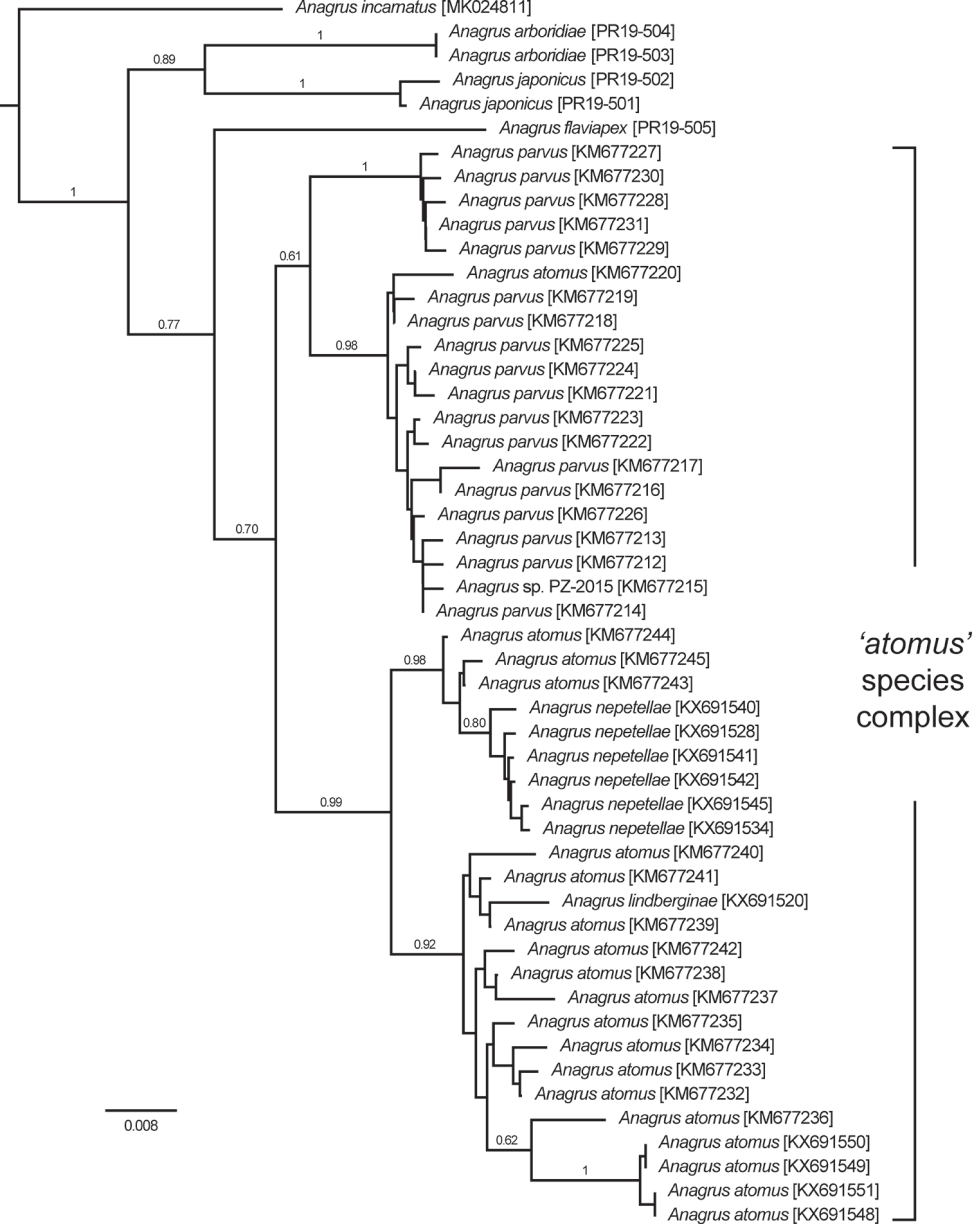
Relationship of *Anagrus arboridiae* sp. nov. with other members of the *A. atomus* species group for which reliable DNA sequences are available, based on a 587 bp fragment of COI. Optimal NJ tree with the sum of branch length = 0.34117946. The percentage of replicate trees in which the associated taxa clustered together in the bootstrap test (1000 replicates) are shown next to the branches and the tree is drawn to scale, with branch lengths indicating uncorrected p-distance. Analyses conducted in MEGA 6.06.

Sequences of the COI gene provided strong evidence that *A. arboridiae* is distinct from *A. flaviapex* and also from members of the *A. atomus* species complex for which comparative sequence data are available. The two *A. arboridiae* specimens (both from Unnan, Shimane Prefecture, Honshu Island) shared a single haplotype which, based on uncorrected p-distance, differed from the nearest taxon, *A. japonicus*, by 5%, and from *A. flaviapex* by 7.3% (Fig. 7; Table 1). In turn, these three species were at least 6.4% divergent from previously sequenced members of the *A. atomus* species complex, which in the Palaearctic region includes *A. atomus*, A. (Anagrus) lindberginae Nugnes & Viggiani, A. (Anagrus) nepetellae Viggiani & Nugnes, and A. (Anagrus) parvus Soyka (Fig. 7; Table 1). Intraspecific variation in the cloned ITS2 sequences was minimal and largely restricted to the length of microsatellite repeat regions. In contrast, the five specimens in this study showed clear interspecific differences between *A. arboridiae*, *A. flaviapex*, and *A. japonicus* in both length (~517, ~591 and ~534bp, respectively) and nucleotide constitution (see GenBank accessions MT414958–MT414966).

**Table 1.** Genetic divergence between *Anagrus arboridiae* sp. nov., *A. japonicus*, *A. flaviapex*, and other members of the *A. atomus* species group including those in the *A. atomus* species complex^*^, based on DNA sequences of the mitochondrial COI gene. Diagonal element shows intraspecific variation (only when more than one sequence was considered). Average pairwise uncorrected p-distances calculated using MEGA 6.06.

	*A. arboridiae*	*A. japonicus*	*A. flaviapex*	A. atomus*	*A. incarnatus* ^§^
*A. arboridiae*	0.000				
*A. japonicus*	0.050	0.005			
*A. flaviapex*	0.073	0.074	–		
A. atomus*	0.073	0.071	0.064	0.034	
*A. incarnatus^§^*	0.073	0.078	0.082	0.080	–

^*^Anagrus atomus species complex as considered by Zanolli et al. (2016) and Nugnes et al. (2017); *^§^*outgroup

## Discussion

The results of this study are well within the expected composition of the genera of the parasitoids, as outside of Japan members of both *Anagrus* and *Oligosita* are known to parasitize eggs of other species of *Arboridia*; however, at species level the parasitoids turned out to be mostly different. Besides the above-mentioned *A. turpanicus* (parasitizing eggs of the invasive *A. kakogawana* in Xinjiang, China), which unlike *A. arboridiae* belongs to the *incarnatus* species group of the nominate subgenus of *Anagrus* (Hu and Triapitsyn 2016), A. (Anagrus) atomus (L.) from the *atomus* species group of the same subgenus had been recorded in Khorasan Province of Iran from eggs of *Arboridia kermanshah*, along with an *Oligosita* sp. (Triapitsyn 1998), and later was studied by Hesami et al. (2004, 2009). Indeed, *Oligosita pallida* had been previously reported as a very effective natural enemy of the same host leafhopper in the Iranian province of West Azerbaijan in the absence of insecticide treatments (Mostaan and Akbarzadeh 1995). Both *A. atomus* and *O. pallida* were also recorded as egg parasitoids of A. (Arboridia) adanae in Turkey (Viggiani 1987; Yiğit and Erkiliç 1987). An *Oligosita* sp. was identified in Turkmenistan from eggs of *Arboridia* sp. on grape (Triapitsyn 1998); it is shown here to belong to *O. pallida* parasitizing eggs of A. (Arboridia) hussaini. Because taxonomy of the speciose and very difficult genus *Oligosita* is in flux worldwide, few previous species identifications can be trusted beyond the generic ones and thus most, especially those outside of Europe, need confirmation.

Of note is the fact that in Japan, most of the obtained *Oligosita* spp. from Shimane Prefecture emerged from table grape leaves collected in non-organic vineyards where no *A. arboridiae* were present, whereas in the organic vineyard in the same prefecture *A. arboridiae* was abundant while only one specimen of *O. pallida* was collected. That perhaps could be due to the longer developmental times of *Oligosita* spp. versus *Anagrus* spp. and the fact that the former spin a cocoon at pupal stage and the latter do not (Chiappini 2002), thus presumably making the immature stages of these trichogrammatids more resistant to insecticides. That assumption, however, needs to be further investigated by a thorough experimental work.

These results could be of importance for other agricultural crops as well because *A. apicalis* is also known to feed on very common, aforementioned fruit trees in Japan. Furthermore, knowledge of the egg parasitoids of *Arboridia* spp. in Japan would be important for the potential classical biological control programs against *A. kakogawana* in other countries of Asia and Europe within the Palaearctic region, where it has established recently as an invasive pest of grapes (Chireceanu et al. 2019).

## Supplementary Material

XML Treatment for Anagrus (Anagrus) arboridiae

XML Treatment for Oligosita pallida

XML Treatment for Oligosita

XML Treatment for Aphelinoidea (Aphelinoidea)
